# Prognostic value of lymph node ratio in children with wilms tumor: a systematic review and meta-analysis

**DOI:** 10.3389/fonc.2026.1890109

**Published:** 2026-10-08

**Authors:** Junyu Dong, Jingwen Ni, Lele Li, Kenan Fang

**Affiliations:** PICU of Luoyang Maternal and Child Health Hospital, Luoyang, China

**Keywords:** children, lymph node ratio, meta-analysis, overall survival, prognosis, Wilms tumor

## Abstract

**Background:**

Lymph node (LN) involvement is a critical determinant of staging and prognosis in Wilms tumor (WT). However, conventional binary nodal staging (positive vs. negative) is often compromised by significant variability in surgical lymph node sampling. The lymph node ratio (LNR), which integrates metastatic burden and sampling adequacy, has emerged as a potential prognostic alternative. This systematic review and meta-analysis evaluated whether LNR serves as an independent predictor of survival outcomes in children with WT.

**Methods:**

A systematic query of PubMed, MEDLINE, Embase, Web of Science, the Cochrane Central Register of Controlled Trials (CENTRAL), and ClinicalTrials.gov was executed, covering the period from their earliest records through May 10, 2026. Retrospective cohort studies reporting hazard ratios (HRs) or risk ratios (RRs) for survival outcomes based on LNR in children with WT were included. Overall survival (OS) was the primary outcome; 5-year, 10-year, and 20-year OS were secondary outcomes. Prespecified subgroup analyses were performed by LNR cutoff (≥0.40 vs. <0.40).

**Results:**

Six retrospective cohort studies involving 4,190 patients met the inclusion criteria. High LNR was significantly associated with poorer overall survival (HR = 2.10; 95% CI: 1.67–2.64). This prognostic significance persisted across secondary outcomes, including 5-year OS (RR = 1.14; 95% CI: 1.11–1.17), 10-year OS (RR = 1.19; 95% CI: 1.15–1.23), and 20-year OS (RR = 1.14; 95% CI: 1.10–1.19). Subgroup analysis revealed no significant interaction based on the LNR cutoff (P = 0.74). Sensitivity analyses confirmed the robustness and stability of the pooled findings.

**Conclusions:**

Higher LNR appears to be associated with poorer overall survival in children with WT and may serve as a useful prognostic indicator. By accounting for both nodal disease burden and surgical effort, LNR may provide additional prognostic information beyond conventional nodal status. While LNR represents a valuable adjunct for individualized treatment decisions, prospective studies are required to establish standardized clinical cut-offs and integrate LNR into formal risk-adapted protocols.

**Systematic review registration:**

https://www.crd.york.ac.uk/PROSPERO/, identifier CRD420261396220.

## Introduction

Wilms tumor (WT) is the most common pediatric renal malignancy, representing approximately 6% to 7% of all childhood cancer cases (1, 2). While advances in multimodal therapy—including surgery, chemotherapy, and radiotherapy—have significantly improved the 5-year overall survival (OS) rate to over 90% in many high-income countries (3, 4), outcomes remain suboptimal for patients with advanced-stage disease or unfavorable histology (5). In some resource-limited settings, the OS rate can be as low as 25% to 53% (6). These disparities underscore the ongoing need for accurate prognostic assessment to refine risk stratification and guide individualized treatment strategies for children with high-risk disease.

Lymph node (LN) involvement is a critical prognostic factor and a primary determinant of staging in WT (7). However, conventional staging methods typically rely on the binary presence or absence of metastatic nodes to determine the clinical stage. This absolute assessment faces challenges due to the marked variability in lymph node sampling patterns. LN yield can be influenced by multiple factors, including patient age, tumor size, and institutional surgical volume (i.e., the annual number of Wilms tumor surgeries performed at a given center, which reflects both surgeon experience and institutional expertise). Institutions with higher surgical volumes are more likely to adhere to standardized lymph node sampling protocols, employ more meticulous surgical techniques, and have greater awareness of the prognostic importance of adequate nodal retrieval-factors that collectively contribute to higher lymph node yields. Conversely, lower-volume centers may have less familiarity with recommended sampling templates, potentially leading to under-sampling and inaccurate staging. In large database studies, median lymph node yields range from only three to five nodes (8). Inadequate node retrieval may lead to potential understaging and has been associated with poorer survival outcomes, thereby compromising the reliability of conventional nodal status as a standalone survival predictor (9).

The lymph node ratio (LNR), calculated as the number of metastatic lymph nodes divided by the total number of examined lymph nodes, has emerged as a compelling alternative index to address these constraints (8). By synthesizing regional disease load with the thoroughness of histological assessment, this metric facilitates more uniform risk categorization across diverse surgical and clinical standards (8, 10). Several retrospective studies have explored the prognostic value of LNR in WT, reporting varying optimal cutoffs (e.g., 0.22, 0.38, 0.40, 0.65) and consistently demonstrating that higher ratios are associated with worse survival (8, 10–12). Although prior pooled analyses have established the LNR as an autonomous predictive marker for survival in multiple tumors, including colorectal cancer (13), non-small cell lung cancer (14), its prognostic significance in children with WT remains unclear.

Given the clinical need for robust prognostic tools, clarifying the collective prognostic significance of LNR in this population is of considerable importance. To address this gap, we conducted a systematic review and meta-analysis to evaluate the predictive value of LNR and to determine whether it independently predicts survival outcomes in children with WT.

## Methods

### Protocol and reporting standard

This systematic review and meta-analysis was conducted in strict accordance with the Preferred Reporting Items for Systematic Reviews and Meta-Analyses (PRISMA) 2020 statement (15). The study protocol was prospectively registered in the International Prospective Register of Systematic Reviews (PROSPERO) under registration number [CRD420261396220]. All study procedures were performed according to prespecified methodological standards to ensure transparency, reproducibility, and methodological rigor.

### Information sources and search strategy

A thorough bibliographic search was executed across PubMed, MEDLINE, Embase, Web of Science, the Cochrane Central Register of Controlled Trials (CENTRAL), and ClinicalTrials.gov from their respective dates of inception through May 10, 2026. To ensure completeness, we also manually screened the reference lists of eligible studies and relevant review articles. In addition, grey literature, preprint records, and online-ahead-of-print publications were considered where applicable. No restrictions on language, country, or geographic region were applied.

The search strategy combined controlled vocabulary terms and free-text keywords related to Wilms tumor (WT), nephroblastoma, lymph node ratio (LNR), lymph node density (LND), positive lymph node ratio, lymph node metastasis, overall survival, and prognosis. The study selection process is summarized in **Figure 1**.

**Figure 1:**
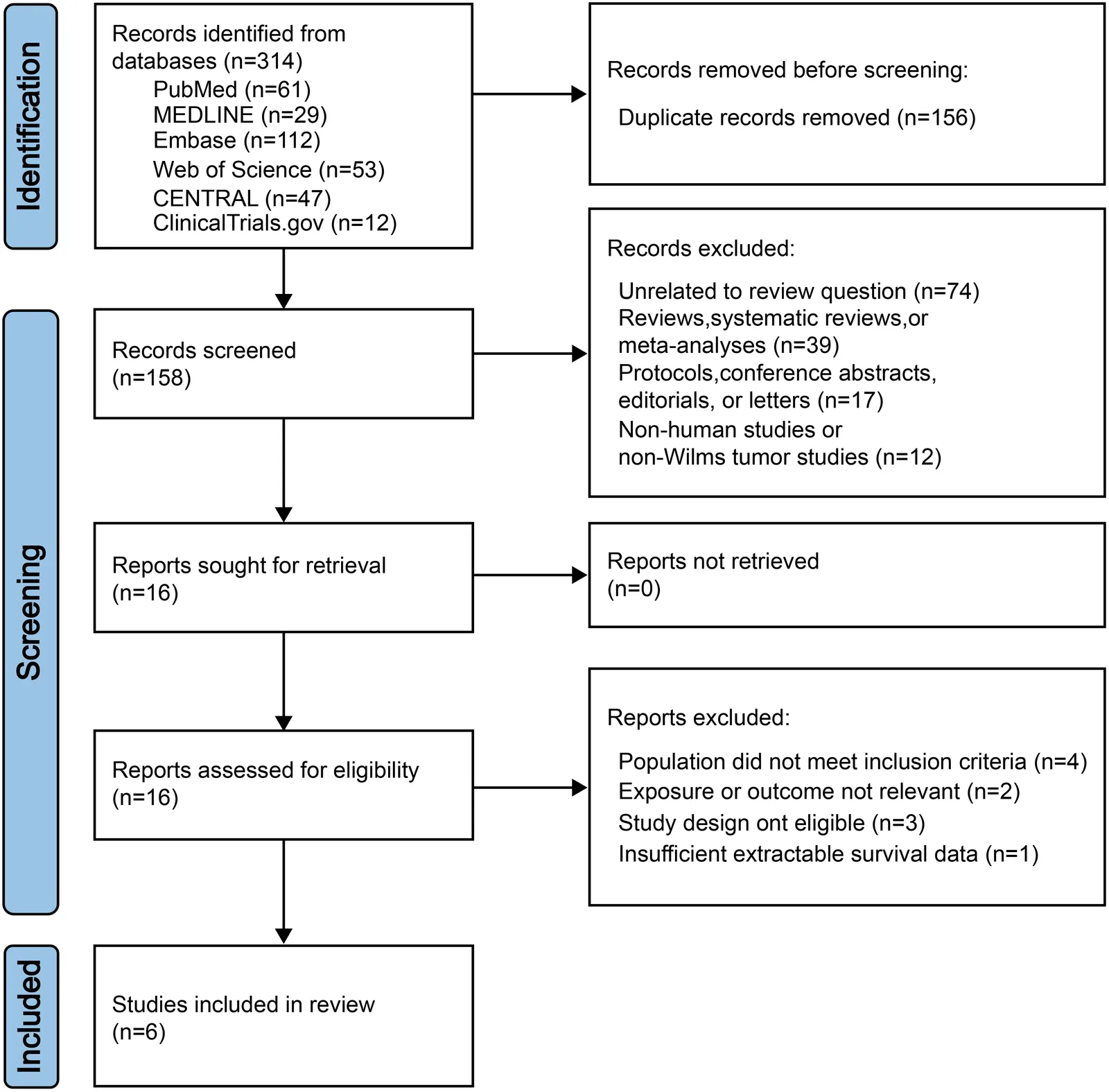
Flow diagram for study selection.

### Eligibility criteria

Eligible studies were selected according to the PECOS (Population, Exposure, Comparator, Outcome, Study Design) framework.

Population: children with histologically confirmed WT.

Exposure: Lymph node ratio (LNR), defined as the ratio of positive or metastatic lymph nodes to the total number of examined lymph nodes. Some eligible studies used the term “lymph node density (LND)” to describe this same ratio. Because all six included studies defined the metric using the same underlying formula, we considered these measures comparable for data synthesis and standardized the terminology as LNR throughout the present meta-analysis.

Comparator: comparison between high and low LNR/LND groups according to study-specific cut-off values.

Outcomes: the primary outcome was overall survival (OS), and the secondary outcomes included 5-year, 10-year, and 20-year overall survival.

Study design: retrospective or prospective cohort studies reporting hazard ratios (HRs) for overall survival or risk ratios (RRs) for time-specific survival outcomes, with corresponding 95% confidence intervals (CIs), or providing sufficient data for their estimation. Reviews, editorials, letters, case reports, conference abstracts without usable data, and studies lacking extractable survival estimates were excluded.

### Study selection

JD, JN, and LL independently screened the titles and abstracts of all records identified through the systematic search. Full texts of potentially eligible studies were then reviewed to determine final inclusion. Any discrepancies were resolved through discussion. If consensus could not be reached, KF (the corresponding author and senior reviewer), who has extensive methodological expertise in systematic reviews and overall responsibility for the study design and supervision, made the final decision. This role was assigned to ensure independence and consistency in resolving unresolved disagreements.

### Data extraction

JD, JN, and LL independently extracted data using a standardized data extraction form. The following variables were collected from each study: first author, publication year, country, sample size, age, sex distribution, tumor type, follow-up duration, definition of LNR and terminology used in the original study, cut-off value, and reported survival outcomes.

For survival analyses, multivariable-adjusted HRs for overall survival and RRs for time-specific survival outcomes, together with corresponding 95% CIs, were preferentially extracted whenever available. If both univariable and multivariable estimates were reported, the multivariable results were used for the primary meta-analysis. All extracted data were checked by KF to ensure completeness and accuracy. Any disagreements were resolved through discussion, with adjudication by KF (the corresponding author and senior reviewer) when necessary. We acknowledge that the included studies used varying LNR/LND cutoff values, and that one study (Chen et al.) (16) employed a cutoff of 0.03, which effectively mirrors binary nodal status within that specific dataset. This issue is further discussed in the limitations.

### Risk of bias assessment

The methodological quality of the included cohort studies was independently assessed by JD, JN, and LL using the Newcastle–Ottawa Scale (NOS) (17). This tool evaluates study quality across three domains: selection of study participants (maximum 4 points), comparability of cohorts (maximum 2 points), and outcome assessment (maximum 3 points), for a maximum total score of 9 points. Studies scoring 7–9 points were considered high quality, those scoring 5–6 points were classified as moderate quality, and those scoring < 5 points were considered low quality. Any disagreements were resolved through discussion, and KF (the corresponding author and senior reviewer) made the final judgment when required.

### Statistical analysis

Statistical analyses were performed using Review Manager (RevMan, version 5.4.2; The Cochrane Collaboration, Copenhagen, Denmark). For the primary outcome of overall survival, hazard ratios (HRs) with 95% confidence intervals (CIs) were synthesized using the generic inverse-variance method. This choice was based on the fact that all included studies reported time-to-event data for overall survival using multivariable Cox proportional hazards models, making HR the most appropriate effect measure for pooling. For secondary outcomes (5-year, 10-year, and 20-year OS), the original studies reported these as binary outcomes (alive vs. deceased) at specific time points. Accordingly, we extracted or calculated risk ratios (RRs) and pooled them using the same inverse-variance method. The risk ratio compares the risk of mortality in the high LNR group relative to the low LNR group. An RR > 1 indicates an increased risk of death associated with a high LNR, an RR < 1 indicates a reduced risk, and statistical significance is determined by whether the 95% confidence interval excludes 1.0.

Statistical heterogeneity was assessed using Cochran’s Q test and quantified with the I^2^ statistic. An I^2^ value <25% was considered low heterogeneity, 25%–50% moderate heterogeneity, 50%–75% substantial heterogeneity, and >75% considerable heterogeneity (18). A random-effects model was applied when significant heterogeneity was present; otherwise, a fixed-effect model was used. A two-sided P value <0.05 was considered statistically significant.

### Subgroup analysis

Prespecified subgroup analyses were conducted to explore potential sources of heterogeneity in the association between LNR and survival outcomes. Studies were stratified according to the cutoff used to define high versus low LNR in the original studies, specifically ≥0.40 versus <0.40. The threshold of 0.40 was prespecified as an exploratory subgroup boundary to facilitate comparison across studies with different LNR cutoffs and was not intended to represent a clinically validated or universally applicable threshold. The primary meta-analysis retained the study-specific LNR cutoffs reported in the original studies. Given the variability in LNR definitions and cutoff values across the included studies, no single clinical cutoff was prespecified as the definitive threshold for patient-level risk classification.

### Sensitivity analysis

Sensitivity analyses were performed to evaluate the robustness of the pooled results. These included reanalysis using a fixed-effect model and leave-one-out analysis, in which each study was sequentially excluded to assess its influence on the pooled estimate.

### Publication bias

Because fewer than 10 studies were available for the primary outcome, formal assessment of publication bias using funnel plots or statistical tests was not performed. Nevertheless, the risk of publication bias was minimized through broad database coverage and manual screening of reference lists.

## Results

### Study selection and characteristics

The comprehensive literature search yielded 314 records across the targeted databases. After the removal of 156 duplicates, 158 unique studies were screened based on titles and abstracts, resulting in the exclusion of 142 papers. A total of 16 articles underwent full-text eligibility assessment, from which six retrospective cohort studies met all inclusion criteria (**Figure 1**). The included studies (8, 10–12, 16, 19), published between 2018 and 2025, provided data for a combined cohort of 4,190 pediatric patients with WT. The six included studies were those by Saltzman et al. (2018) (8), You et al. (2018) (10), Pan et al. (2019) (12), Walker et al. (2020) (11), Chen et al. (2024) (16), and Ziogas et al. (2025) (19). Individual sample sizes ranged from 231 to 1,924 participants. The mean age of the included populations was relatively consistent, typically between 3.00 and 4.00 years. LNR cut-off values demonstrated variability across the literature, with thresholds including 0.03, 0.2, 0.22, 0.38, 0.4, and 0.65. Quality evaluation via the Newcastle–Ottawa Scale (NOS) indicated that all studies were of moderate to high quality, with scores ranging from 6 to 8. Detailed characteristics and quality metrics are presented in **Tables 1**, **2**.

**Table 1:** Main characteristics of the included literature.

First author, year	Sample size	Research design	Age	Sex(female:male)	Cancer type	Histology/predominant histologic component	Follow-up	Reported LNR cutoff	NOS score
Chen et al. (16)	874	cohort study	3.40 ± 2.82	462:412	Wilms tumor	NR	5 years	0.03	8
Pan et al. (12)	376	cohort study	4.00 ± 2.20	211:165	Wilms tumor	NR	N/A	0.65	7
Saltzman et al. (8)	390	cohort study	4.00 ± 2.20	223:167	Favorable histology Wilms tumor	Favorable histology; predominant component NR	5 years	0.38	6
Walker et al. (11)	231	cohort study	4.00 ± 2.22	122:109	Wilms tumor	NR	5 years	0.4	8
You et al. (10)	1924	cohort study	3.00 ± 2.20	1007:917	Wilms tumor	NR	N/A	0.22	8
Ziogas et al. (19)	395	cohort study	3.00 ± 3.00	213:182	Wilms tumor	NR	N/A	0.2	8

LNR, lymph node ratio; NOS, Newcastle-Ottawa Scale; NR, not reported. Predominant histologic component (blastemal, epithelial, or mesenchymal) was not reported in any of the included studies, as the SEER and NCDB registries from which these cohorts were derived do not capture the proportional distribution of these three components; they primarily record a binary classification of favorable versus unfavorable histology based on the presence or absence of anaplasia.

**Table 2:** Quality of the included studies.

Study	Representativeness	Selection of non-exposed	Ascertainment of exposure	Outcome not present at start	Comparability (main factors)	Comparability (other factors)	Assessment of outcome	Long enough follow-up	Adequacy of follow-up	Total
Chen	✓	✓	✓	✓	✓	✓	✓	×	✓	8
Pan	✓	×	✓	✓	✓	✓	✓	×	✓	7
Saltzman	✓	×	✓	✓	✓	✓	×	×	✓	6
Walker	✓	✓	✓	✓	✓	✓	✓	×	✓	8
You	✓	✓	✓	✓	✓	✓	✓	×	✓	8
Ziogas	✓	✓	✓	✓	✓	✓	✓	×	✓	8

### Primary outcome

Six studies (8, 10–12, 16, 19) reported OS. The pooled analysis indicated that patients in the high LNR group had a significantly poorer overall survival compared to those in the low LNR group (HR = 2.10; 95%CI: 1.67–2.64; I^2^ = 0; P < 0.00001) (**Figure 2**). To ensure the stability and reliability of the primary findings, sensitivity analyses were conducted. The pooled HR remained robust when comparing fixed-effect models. Furthermore, a leave-one-out sensitivity analysis demonstrated that no single study disproportionately influenced the results, with pooled HRs ranging from 2.02 to 2.19, all of which remained statistically significant. These findings are summarized in **Table 3**. Prespecified subgroup analysis stratified studies according to whether their reported LNR cutoff was < 0.40 or ≥ 0.40. In studies using a cutoff < 0.40, the pooled HR was 2.15 (95% CI 1.65–2.79), whereas in studies using a cutoff ≥ 0.40, the pooled HR was 1.97 (95% CI 1.24–3.12). There was no significant interaction between the two subgroups (P = 0.74; I^2^ = 0%). (**Figure 3**).

**Figure 2:**
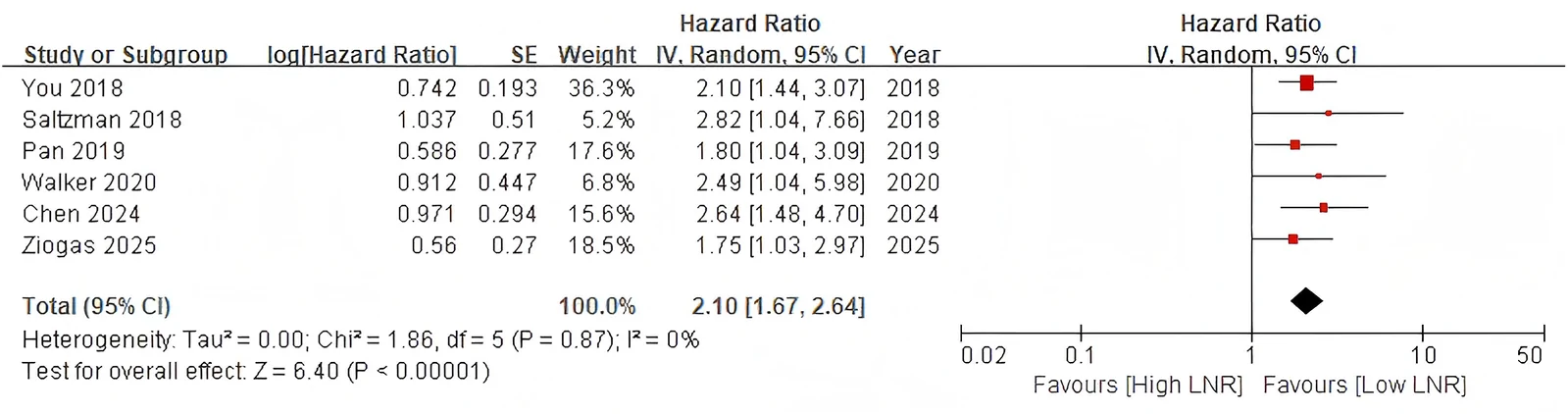
Forest plot comparing overall survival between high and low lymph node ratio groups in children with Wilms tumor.

**Table 3:** Sensitivity analysis of meta-analysis.

Analysis	NO. patients(trials)	HR	95%CI
All trials	4190(6)	2.10	1.67, 2.64
Using fixed-effect models	4190(6)	2.10	1.67, 2.64
Excluding each study in turn			
Excluding Saltzman et al., 2018 (8)	3800(5)	2.07	1.64, 2.62
Excluding Pan et al., 2019 (12)	3814(5)	2.18	1.69, 2.80
Excluding Walker et al., 2020 (11)	3959(5)	2.08	1.64, 2.63
Excluding Chen et al., 2024 (16)	3316(5)	2.02	1.57, 2.58
Excluding You et al., 2018 (10)	2266(5)	2.10	1.58, 2.80
Excluding Ziogas et al., 2025 (19)	3795(5)	2.19	1.70, 2.82

HR, Hazard Ratio; CI, confidence interval.

**Figure 3:**
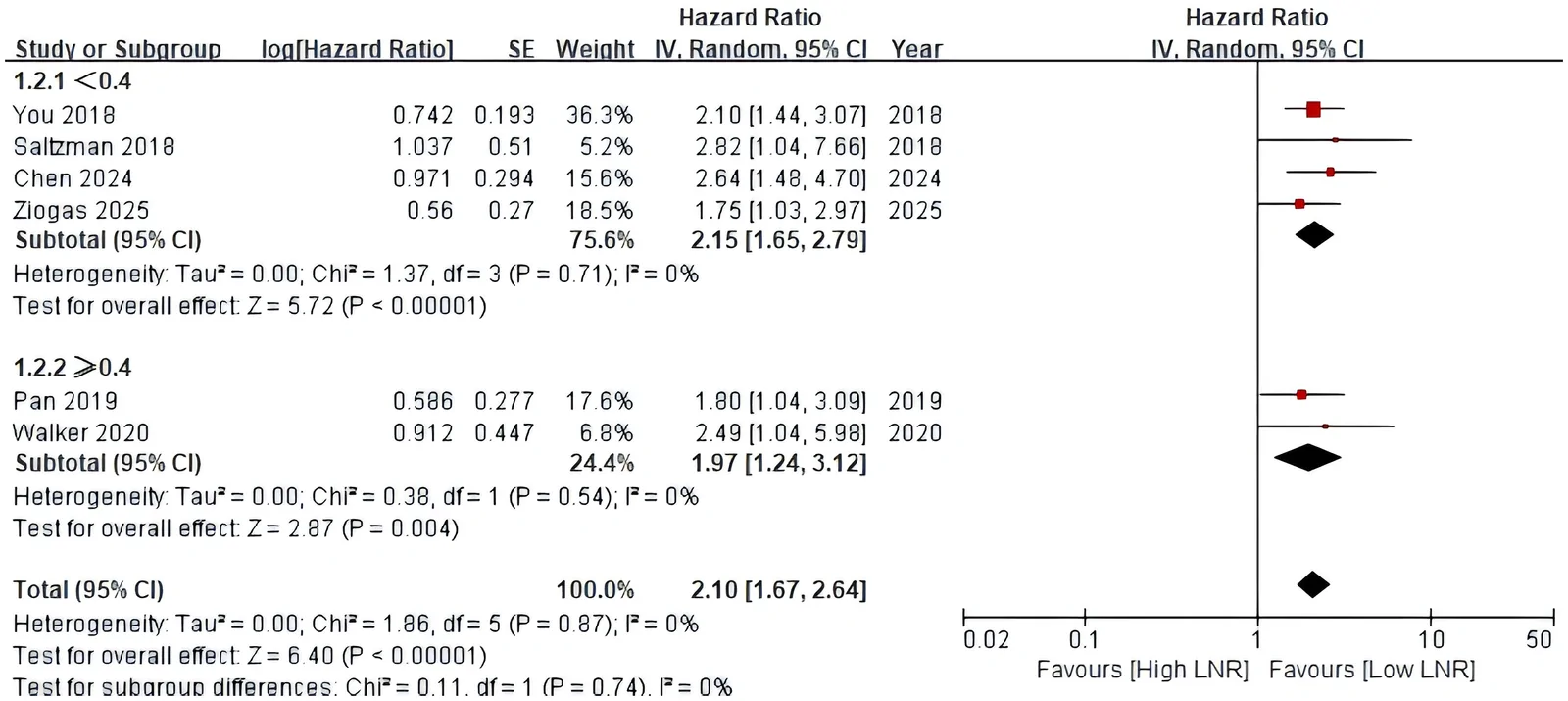
Subgroup analysis of overall survival according to study-specific LNR cutoffs categorized as <0.40 or ≥0.40 in children with Wilms tumor.

### Secondary outcome

The secondary outcomes consistently demonstrated the prognostic significance of the LNR across various survival intervals. Among the five studies (8, 10–12, 16) reporting 5-year OS, two studies (10, 12) with a combined cohort of 2,300 patients also provided data on 10-year and 20-year OS. Specifically, five studies encompassing 3,323 patients showed that a high LNR was significantly associated with poorer 5-year OS (RR = 1.14; 95% CI: 1.11–1.17; I^2^ = 0; P < 0.00001). This trend persisted in longer-term follow-up; data from two studies involving 2,300 patients revealed a pooled RR of 1.19 (95% CI: 1.15–1.23; I^2^ = 0; P < 0.00001) for 10-year OS, and a pooled RR of 1.14 (95% CI: 1.10–1.19; I^2^ = 0; P < 0.00001) for 20-year OS based on the same cohort size (**Figures 4**–**6**).

**Figure 4:**
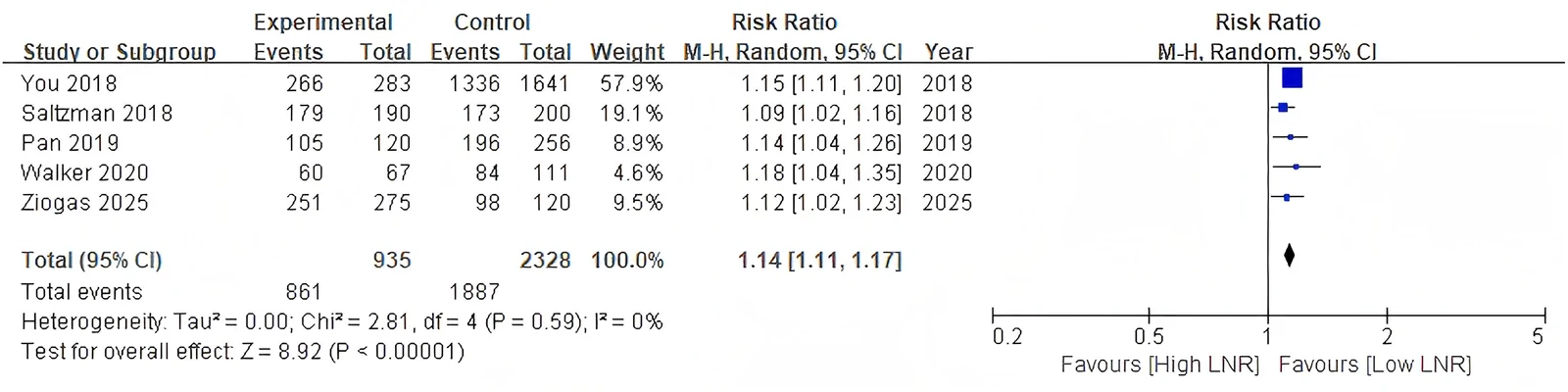
Forest plot comparing 5-year overall survival between high and low lymph node ratio groups in children with Wilms tumor.

**Figure 5:**
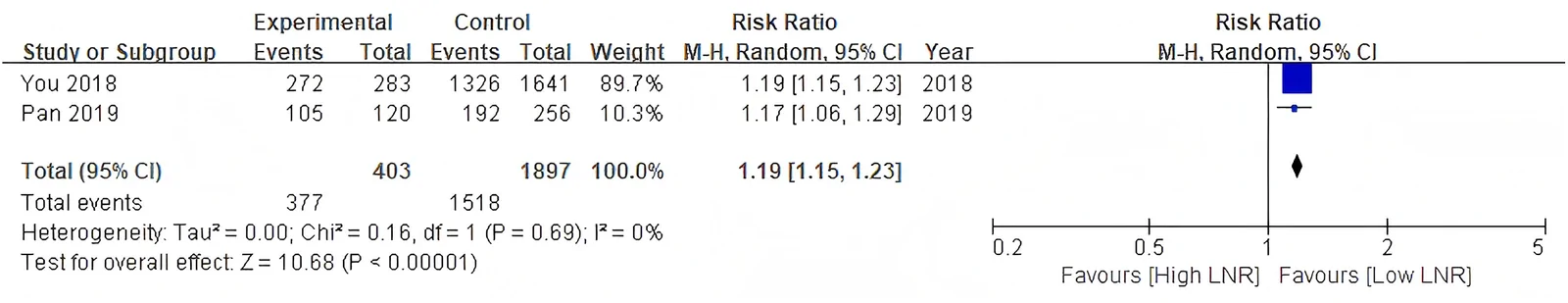
Forest plot comparing 10-year overall survival between high and low lymph node ratio groups in children with Wilms tumor.

**Figure 6:**
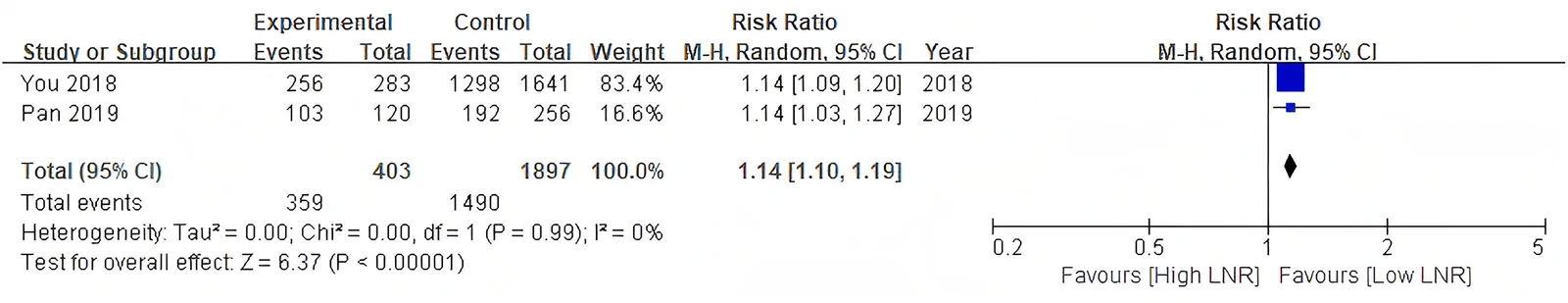
Forest plot comparing 20-year overall survival between high and low lymph node ratio groups in children with Wilms tumor.

## Discussion

This systematic review and meta-analysis synthesized data from six retrospective cohort studies (8, 10–12, 16, 19) investigating the prognostic role of the LNR in pediatric patients with WT. Across all included cohorts, we observed that a higher LNR was consistently and significantly associated with an increased risk of mortality and inferior OS.

To our knowledge, this meta-analysis provides systematic evidence that an elevated LNR serves as a significant and independent prognostic marker for survival in children with WT, a finding that aligns with previous research across various solid malignancies (14, 20, 21). The inherent superiority of LNR lies in its capacity to mitigate the heavy reliance on sampling adequacy that often compromises traditional staging systems. In clinical practice, the consistency of lymph node retrieval in WT varies significantly, with reported omission rates as high as 9% to 42% (11, 19). Under such suboptimal sampling conditions, relying solely on absolute positive lymph node counts increases the risk of false-negative results and ‘stage migration’ which inevitably leads to under-staging and insufficient postoperative therapy (7, 9). Notably, a low LNR does not necessarily indicate favorable prognosis in the context of extremely inadequate nodal sampling; if only one negative node is retrieved, LNR would be zero, potentially yielding a false sense of security. The mathematical relationship between lymph node yield and LNR warrants careful consideration. With a small denominator (e.g., 1–3 nodes), a single positive node yields a disproportionately high LNR, potentially overestimating disease burden. Conversely, with a larger denominator (e.g., ≥ 10 nodes), the same positive node count yields a substantially lower LNR, providing a more stable estimate of regional spread. While Kieran et al. (7) suggested that examining at least 7 nodes reduces relapse risk, and Saltzman et al. (8) proposed that 6–10 nodes (or a minimum of 10 nodes) may optimize staging accuracy, no universally accepted minimum threshold has been formally established in pediatric renal oncology. In the six large registry-based cohorts included in this meta-analysis, the median lymph node yield consistently ranged from only 3 to 5 nodes across different studies (8, 10–12, 19), and Walker et al. (11) specifically reported that 21.1% of patients had no or unknown lymph node sampling. This indicates that current surgical practice often falls short of recommended targets. Therefore, LNR interpretation should always account for the adequacy of nodal sampling. Importantly, we acknowledge that our meta-analysis, being based on aggregated registry data, could not perform a subgroup analysis stratified by lymph node yield to assess whether the prognostic value of LNR varies with sampling adequacy. This represents a significant limitation and underscores the urgent need for prospective studies to define the minimum number of examined nodes required for reliable LNR calculation in Wilms tumor, as well as to validate standardized LNR cutoffs within future risk-adapted treatment protocols. Thus, LNR is best applied when a minimum number of examined lymph nodes is achieved, a threshold that remains to be prospectively defined in pediatric renal oncology (22). Consequently, this proportional metric offsets the biases introduced by inadequate node retrieval, offering a more stable and authentic representation of regional disease spread than conventional staging methods (8, 11, 19).

The clinical implications of our results are significant. High LNR identifies a subgroup of WT patients at markedly elevated risk who may benefit from intensified postoperative regimens, such as the addition of doxorubicin or abdominal radiation in appropriately selected high-risk settings (23, 24). Conversely, LNR might help identify low-risk individuals eligible for therapy reduction to minimize long-term toxicities, such as doxorubicin-induced cardiotoxicity (25). Our results underscore the urgent need for standardized, template-based lymph node sampling during nephrectomy to maximize staging accuracy and disease control (26). Future research should prioritize prospective validation of LNR within standardized treatment protocols, such as COG or SIOP, and explore the integration of LNR with molecular markers to refine multivariate prognostic models (24, 26).

This meta-analysis has several limitations. First, all included studies were retrospective analyses of large administrative databases (such as SEER and NCDB), which carry inherent risks of selection bias and missing data (27, 28). Second, there was substantial variability in the reported LNR cutoff values across studies, ranging from 0.03 to 0.65. Importantly, the 0.40 threshold used in our subgroup analysis was a prespecified exploratory boundary rather than a clinically validated cutoff, and the primary meta-analysis retained the study-specific thresholds reported by the original studies. Although several studies identified thresholds in the range of 0.20–0.40, the available aggregate-level evidence is insufficient to establish a single clinically applicable LNR cutoff for pediatric WT. Future research should evaluate the continuous relationship between LNR and survival using individual-participant-level data, nonlinear modeling, or time-to-event receiver operating characteristic analyses, followed by prospective validation of candidate thresholds within standardized treatment protocols. In particular, the cutoff of 0.03 used in the Chen et al. (2024) (16) study effectively served as a proxy for binary nodal status within that cohort, as no patient in their dataset exhibited a single positive node with a total examined node count high enough (e.g., ≥ 34 nodes) to generate an LND between 0.03 and 1.0. Consequently, this specific cutoff represents a statistical outlier that replicates conventional nodal positivity rather than functioning as a genuine graded ratio metric. Although this study technically met our predefined inclusion criteria based on explicit LND reporting, this observation highlights the substantial heterogeneity in cutoff selection. Importantly, excluding the Chen et al. (16) study in our sensitivity analysis did not materially alter the pooled effect estimate (HR = 2.02; 95% CI: 1.57–2.58), confirming the robustness of our primary conclusion. Future studies should prioritize the establishment of standardized LNR thresholds using individual-participant-level data. Rather than relying solely on study-specific dichotomization, LNR should initially be evaluated as a continuous variable to characterize its linear or nonlinear association with survival. Candidate thresholds could then be derived using time-to-event receiver operating characteristic analyses, maximally selected rank statistics, or clinically anchored risk stratification methods. These thresholds should be assessed not only for discrimination but also for calibration and clinical utility, and subsequently validated prospectively in independent cohorts. Individual-participant-data meta-analysis would be particularly valuable for determining whether a common threshold is appropriate across different patient populations, surgical practices, and treatment protocols. Third, most datasets lacked detailed granularity regarding tumor histology (e.g., presence of anaplasia) and molecular biomarkers (e.g., 1p/16q loss of heterozygosity), which are critical for current risk-adapted protocols (24, 29). WT is a histologically heterogeneous neoplasm composed of varying proportions of blastemal, epithelial, and stromal components. The prognostic implications of these histological components depend, at least in part, on the treatment context. Under the SIOP histopathological classification for patients treated with preoperative chemotherapy, blastemal-type WT is classified as high risk, whereas epithelial-, stromal-, mixed-, and regressive-type tumours are generally classified as intermediate risk (30). However, these associations primarily reflect differences in prognosis and treatment response across histological subtypes, and whether any predominant histological component independently confers an increased propensity for lymph-node metastasis remains uncertain. Moreover, preoperative chemotherapy can substantially alter the relative proportions of residual viable histological components, making direct comparisons of predominant histological patterns across different treatment strategies challenging. Notably, none of the studies included in this meta-analysis provided extractable data on the relative proportions or predominant patterns of blastemal, epithelial, and stromal components, nor did they report lymph-node involvement or LNR-associated survival outcomes according to predominant histological composition. Saltzman et al. (8) included only patients with favourable-histology WT; however, this classification primarily reflects the presence or absence of anaplasia rather than the predominance of a specific histological component. Consequently, the available evidence does not allow us to determine whether histological composition is associated with a propensity for lymph-node metastasis or whether it modifies the prognostic association between LNR and survival outcomes. Future prospective studies should systematically and standardly report histological composition, anaplastic status, timing of treatment, lymph-node involvement, and LNR to clarify the interplay between tumour histology, lymphatic dissemination, and the prognostic value of LNR. Anaplasia, defined as the presence of large, hyperchromatic nuclei and multipolar mitotic figures, is a well-established adverse prognostic factor in WT and constitutes the primary basis for unfavorable histology classification (31). However, the relationship between anaplasia and lymph node metastasis remains uncertain. While some evidence suggests an increased risk of lymphatic spread in anaplastic tumors, other reports have found no independent association after adjusting for stage and other confounders (29). Importantly, anaplasia and lymph node involvement are generally considered independent prognostic factors in current risk-stratification systems (24), suggesting that each contributes separately to outcome prediction. In our meta-analysis, none of the included studies reported extractable data on the interaction between anaplasia status and LNR or provided anaplasia-specific hazard ratios. Consequently, we could not assess whether the prognostic value of LNR differs by anaplasia status. This represents an important limitation and a priority for future research, particularly given that diffuse anaplasia is a key determinant of treatment intensification in current COG protocols. Furthermore, lymph node assessment in all included studies relied exclusively on conventional histopathological examination. To our knowledge, no studies have investigated lymph node involvement in WT using molecular techniques such as one-step nucleic acid amplification (OSNA), which detects cytokeratin 19 mRNA as a marker of epithelial tumor cells and has been validated for lymph node staging in breast (2) and colorectal cancers (32). The absence of molecular lymph node assessment in WT represents a significant knowledge gap. While OSNA has not yet been applied to pediatric renal tumors, its potential to detect micrometastases with higher sensitivity than conventional histology could refine nodal staging and potentially impact risk stratification, particularly for patients who might be candidates for therapy de-escalation. Future prospective studies should explore the feasibility and clinical utility of molecular lymph node analysis in WT, especially given the prognostic importance of nodal status and the need to minimize long-term treatment-related toxicities. Fourth, a direct meta-analytic comparison between LNR and binary nodal status could not be performed because not all included studies reported extractable multivariable-adjusted hazard ratios for binary nodal status. Thus, readers may question whether LNR truly offers clinical advantages over conventional nodal classification. However, prior studies indicate that LNR provides incremental value: Saltzman et al. (8) showed that among node-positive patients, LNR further stratified survival risk, whereas binary status alone could not; Walker et al. (11) found that LNR better accounts for sampling adequacy and reduces understaging; and Kieran et al. (7) emphasized that the number of positive nodes carries prognostic significance beyond mere positivity. These observations suggest LNR adds prognostic granularity beyond binary nodal status. Nevertheless, formal head-to-head validation within a meta-analytic framework remains a priority for future prospective research. Fifth, terminology for the lymph node ratio has varied across the literature, with some studies referring to the same metric as “lymph node density (LND)”. In all six studies included in this meta-analysis, the metric was calculated using the same underlying formula, namely metastatic lymph nodes divided by the total number of examined lymph nodes. To avoid terminological ambiguity, we have standardized the terminology as “lymph node ratio (LNR) “ throughout the present meta-analysis, while acknowledging that “LND” was used in some of the original publications. Sixth, the included studies lacked detailed information on histological composition. None of the original studies reported the relative proportions or predominant patterns of blastemal, epithelial, and stromal components in an extractable form. Consequently, we could not determine whether predominance of a specific histological component was associated with a greater propensity for lymph-node metastasis or modified the prognostic association between LNR and survival outcomes. Prospective studies incorporating standardised histopathological reporting, with systematic documentation of histological composition, lymph-node status, and LNR, are warranted to further elucidate the relationships among these factors.

## Conclusion

Higher LNR may be an important prognostic indicator of poorer overall survival in children with WT. It provides superior risk stratification beyond binary nodal status by accounting for both metastatic burden and surgical adequacy. While LNR may provide useful prognostic information beyond conventional nodal status, prospective studies are needed to establish and validate standardized clinical cutoffs before LNR can be incorporated into routine risk-adapted decision-making.

## Data Availability

The original contributions presented in the study are included in the article/supplementary-material. Further inquiries can be directed to the corresponding author.
